# Insect meals in cat diets and their effects on digestibility, physiology, and gut microbiota

**DOI:** 10.3389/fvets.2025.1592625

**Published:** 2025-05-29

**Authors:** Karen Guttenkunst Lisenko, Flavia Maria de Oliveira Borges Saad, Maiara Rodrigues Duarte Oliveira, Thaiane Vieira da Silva, Daniel Souza Dias, Luiz Duarte de Oliveira, Sudário Roberto Silva Júnior, Júlio Cézar dos Santos Nascimento, Apolônio Gomes Ribeiro, Márcio Gilberto Zangeronimo, Diego Vicente da Costa, Lucas Rannier Ribeiro Antonino Carvalho, Maria Regina Cattai de Godoy

**Affiliations:** ^1^Department of Animal Sciences, Federal University of Lavras, Campus Universitário, Lavras, Brazil; ^2^Animal Science Department, Universidade Federal Rural de Pernambuco, Recife, Brazil; ^3^Animal Science Department, Universidade Federal da Paraíba, Areia, Brazil; ^4^Agricultural Sciences Institute, Campus Regional de Montes Claros, Federal University of Minas Gerais, Montes Claros, Brazil; ^5^Department of Physiology and Pharmacology, Stockholm Sweden Biomedicum, Karolinska Institutet, Stockholm, Sweden; ^6^Department of Animal Sciences, University of Illinois, Urbana, IL, United States

**Keywords:** pet nutrition, domestic cat, insects, digestibility, microbiota

## Abstract

Insects are a valuable source of nutrients, but little is known about their nutritional value for companion animals. In this study, we evaluated the inclusion of three insect meals in cat diets (Cinerea cockroach, CC; Madagascar cockroach, MC; and Superworm, SW) at two different levels (7.5 and 15%) on apparent digestibility coefficient (ADC), blood parameters, fecal pH, short-chain fatty acids (SCFA), branched-chain fatty acids (BCFA), phenol and indole production, and gut microbiota during six experimental periods of 15 days each. No differences were found for ADC, except for chitin in which MC registered the highest ADC. The fermentative product analysis showed that propionate displayed higher abundance in all insect treatments compared to the control group. Moreover, cats fed CC diet resulted in higher fecal butyrate while higher 4-methylphenol was registered in cats fed MC and SW diets. No significant differences were found for fecal pH and score, as well as no change in urea, creatinine, and blood count were registered. No differences were registered for total fecal SCFA, BCFA, phenol, and indole production compared to the control group or between insect meal fed groups. The fecal microbiota analyzed by gene 16S rRNA sequencing of cats did not register differences in alpha or beta diversity. In conclusion, dietary inclusion of insect meal up to 15% is a suitable alternative food for adult cats.

## 1 Introduction

The pet food industry has experienced significant growth in recent years, leading to increased demand for high-quality protein resources. This surge has created competition with the human food chain, livestock sector, and fish production systems (1). Traditionally, pet food has relied on various protein sources such as meat and bone meals, poultry by-products, fishmeal, and lamb meal (2). However, finding alternative dietary ingredients is necessary to ensure sufficient food production to meet global demand.

Insect meal production has emerged as a rapidly growing industry, offering a promising alternative source of protein and lipids for animal feed (3, 4). Insects possess several advantages, including rapid growth and reproduction, efficient conversion of bio-waste into protein, and lower energy, land, and water requirements compared to traditional protein sources (4, 5). Additionally, it is worth noting that insects are already a natural part of the diet for many wild cats worldwide, comprising at least 6% of their overall food intake (6, 7).

Among various insect species, larvae of Madagascar cockroach (*Gromphadorhina portentosa*, Blattodea), Cinerea cockroach (*Nauphoeta cinerea*, Blattodea), and Superworm (*Zophobas morio*, Coleoptera), can be reared on a broad variety of organic materials, contributing to their sustainability as feed ingredients. Recent studies have evaluated the use of these insects to feed humans (8–10) and several fish species (11–13), poultry (14–16) and pigs (17). Although insects have been used to feed most farmed animals, few studies have been performed to investigate insect meals as a dietary ingredient for pet animals (6, 18). This gap is even more evident when it comes to domestic cats, for which the available data remain limited.

Therefore, this research aimed to assess the nutritional digestibility, concentration of phenols, indoles, and short-chain fatty acids, as well as blood parameters and fecal microbiota in adult cats fed three types of insect meal: Madagascar cockroach (*Gromphadorhina portentosa*), Cinerea cockroach (*Nauphoeta cinerea*), and Superworm (*Zophobas morio*). This study seeks to contribute to the understanding of the potential benefits of using insect meals as a sustainable dietary option for domestic cat.

## 2 Materials and methods

The experiment was conducted at the Center for Studies on Companion Animal Nutrition (CENAC) in the Department of Animal Sciences at the Federal University of Lavras (UFLA), located in Lavras, Minas Gerais, Brazil. The experimental procedures were approved by the UFLA Ethics Committee on Animal Use, protocol no 072/16.

### 2.1 Animals and facilities

Six male adult cats with no defined breed, weighing 3.72 ± 0.86 kg and approximated 5 years old were used. The animals underwent a clinical examination at the beginning and end of the experiment. Animals were kept in individual enclosures of galvanized wire with 50 × 70 × 60 cm (width × depth × height, respectively), with pet feeders and water fountains in adequate quantity, with suspended rest area and double sanitary trays for the separation of urine and faeces. All cats entered into the study were fed the same reference diet during a 2-week baseline period. Before the trial starts, a control sample of all cat faeces were collected for fecal microbiota analysis.

### 2.2 Experimental diets

The six treatments consisted of three insect meals Madagascar cockroach *Gromphadorhina portentosa*, Cinerea cockroach *Nauphoeta cinerea,* and Superworm *Zophobas morio* at two inclusion levels 7.5 and 15%. The insects were obtained from the Laboratory of Entomoculture at the Institute of Agricultural Sciences of the Federal University of Minas Gerais, Montes Claros, Minas Gerais, Brazil. The insects were reared on a diet consisting of soybean, corn, and wheat, killed by immersion in boiling water, dried in a forced-air oven at 50°C for 48 h, and milled in an electric screw meat grinder. The three insects used were harvested as larvae.

The insect meals were included in partial replacement of a reference diet according to the substitution methodology proposed by Matterson et al. (19). The reference diet was based on two commercial feed (dry and moist feeds) in a 4:1 ratio, respectively. The composition of the experimental diets in this ratio was idealized to ensure high palatability and good homogenization when the insect meals were included in the treatments, in partial replacement of the wet diet. The control diet was used only as a reference diet, and the data collected from this group was used to calculate the test ingredient’s ADCs, as described below. The chemical composition of the insect meals and the diets are presented in Table 1. The amount of feed provided was determined using standard equations for the daily energy requirements of adult cats (110 kcal × kg BW^0.67^), according to recommendations of the National Research Council (U.S.) Committee on Dog and Cat Nutrition NRC (20). The daily amount was divided into two equal meals which were offered at 8:00 h and 17:00 h.

**Table 1:** Nutritional composition (%, DM-basis) and energy value (kcal/kg) of insect meals, reference diet, and experimental diets for adult cats.

Item	Insect meals			Reference diet	Treatments^a^					
	CC	MC	SW		7.5% CC	15% CC	7.5% MC	15% MC	7.5% SW	15% SW
DM	93.96	94.60	94.57	77.89	77.93	78.38	78.43	78.95	79.40	80.18
EE	22.68	12.97	33.05	13.68	12.55	13.59	14.62	16.75	14.66	16.58
CP	64.78	78.87	49.20	34.13	33.53	41.88	37.66	35.21	29.35	38.11
MM	3.68	3.89	2.77	8.14	5.80	5.86	5.70	5.41	5.40	5.23
Chitin	8.68	10.31	8.01	1.12	4.41	5.01	3.36	5.05	2.89	1.68
Energy	5,581	5,362	6,779	4,741	4,742	4,717	4,758	4,860	4,773	4,937

a Formulated diets containing different sources of insect meal at different ratios.

CC = Cinerea Cockroach; MC = Madagascar Cockroach; SW = Superworm, DM = Dry Matter, EE = Ethereal Extract in Acid Hydrolysis, CP = Crude Protein, MM = Mineral Matter.

### 2.3 Experimental procedures and sample collection

The data of apparent digestibility coefficients (ADC) of the reference diet (dry and moist feeds) were obtained by a preliminary trial, carried out for 15 days. The reference diet was administrated to the six cats for ten days for diet adaptation, and faeces collection lasted the following five days. The data on digestibility of the reference diet was used to calculate the digestibility coefficients of the insect meals later evaluated. Then, the experiment was conducted using a Latin square design, in a 3 × 2 factorial scheme (the three insect meals, and the two inclusion levels—7.5 and 15%) with six replicates. All animals received one of the six diets, and diets were alternated in the following period such that all cats received all diets, with six cats per diet. Each period lasted 15 days: from day 1 to 10, cats were adapted to the diets; from day 10 to 12, faeces were collected to determine the apparent digestibility coefficient (ADC) of nutrients; from day 13 to 15, urine was collected simultaneously to the fresh faeces, using cages adapted to collector trays to evaluate fermentation products, urinary pH and density; on day 15, 5 mL blood sample was collected from each cat by cephalic venous puncture. Blood samples were sent to a commercial clinical laboratory for hematological parameters evaluation.

The apparent digestibility coefficient (ADC) of the nutrients, i.e., dry matter, organic matter, crude protein, chitin, lipids, and energy were calculated according to the quantitative faeces collection protocol and calculation procedures described by the Matterson et al. (19). Faeces were collected twice a day, packed in plastic bags, weighted, and stored at −20°C freezer until the end of the sample collection period.

Fecal output and score were evaluated with grades from 1 to 5 and classified according to Carciofi et al. (21): 0 = watery liquid, which can be poured; 1 = unformed and soft stools; 2 = soft, malformed stool; 3 = formed, moist and soft stools; 4 = consistent stool, which does not adhere to the floor; and 5 = dry and hard well-formed stools.

Fecal pH, dry matter (DM), acetate, propionate, and butyrate branched short-chain fatty acids (BSCFAs) concentrations, isovalerate, and isobutyrate, branched-chain fatty acids (BCFAs), valerate, indole, phenol and gut microbiota were measured in fresh faeces, collected within 15 min of defecation of each cat. Fecal pH was determined using a digital pH meter (model Q400A, Quimis, São Paulo, Brazil), the feces were placed directly in contact with the pH electrode and measured. DM was determined according to Latimer (22) analyses. For indole and phenol determination, 5 g of faeces were mixed in 2 N HCl solution in a 1:1 ratio, for determination of BSCFAs and BCFAs. For the fecal microbiota analysis, faeces samples were frozen immediately after collection in liquid nitrogen and kept at −80°C.

In order to preserve the urine sample against microbial action, 0.1 g of thymol was applied to the trays during the sample collection periods. Urinary pH was measured at 8 am using the same digital pH Meter described for fecal pH determination. The urinary density was determined by a portable refractometer (Instrutherm, model RTP – 20ATC, São Paulo, Brazil).

### 2.4 Chemical analysis and apparent digestibility coefficient (ADC) determination

Chemical analysis of the diets and fecal samples were analyzed for dry matter (DM) (method 934.01), crude protein (CP) (method 954.01), and mineral matter (MM) (method 942.05) according to methodologies described by the Association of Official Analytical Chemists (23). The lipid content (EE) was determined by acid hydrolysis followed by ether extraction according to methodologies described by the AOAC (24) and chitin content (C) was measured according to Hornung and Stevenson (25), and Ma and Zuazaga (26). The ADC DM was calculated by the formula: ADC DM (%) = [(a−b)/a] × 100, in which a = feed intake, DM basis and b = faeces excretion, DM basis. The ADCs of dietary nutrients were calculated by the equation: Nutrient ADC (%) = [(a × b – c × d)] / (a × b) × 100, in which a = feed intake, DM basis; b = nutrient percentage in the feed; c = faeces excretion, DM basis; d = nutrient percentage in faeces. The nutrients ADC and AMEDM (Apparent metabolizable energy of dry matter) of insect meals were determined according to Matterson et al. (19): ADC (% or kcal/kg) = ADC bd + ((ADC td–ADC bd)/P), in which bd = basal diet (0%; % in DM); td = test diet (7.5% or 15% in DM); P = substitution percentage of test ingredient.

Fermentative product analysis (BSCFA, BCFAs, indole, and phenol) and fecal microbiota were conducted at Illinois University, Urbana-Champaign, United States. Phenol concentrations and fecal indole were determined using gas chromatography, accordingly to Flickinger et al. (27), methods, while the concentrations of BSCFAs and BCFAs on faeces samples, were determined by gas-phase chromatography, following Erwin et al. (28), using a Hewlett-Packard 5890A chromatograph (Palo Alto, California, United States) series II, and one glass column (180 cm × 4 mm id) containing 10% SP-1200/1% of H_3_PO_4_ in directive 80/100 + Chromosorb WAW mesh (Supelco Inc., Bellefonte, PA). Nitrogen was the carrier with a flow rate coefficient of 75 mL/min, temperatures utilized on the oven, detector, and injector were 125, 175, and 180°C, respectively.

### 2.5 Fecal microbiota: DNA extraction, amplification, sequencing and bioinformatics

Fecal samples were collected before and after each 15-day sample period. Fecal DNA extraction was obtained utilizing the commercial kit Mo-Bio PowerSoil (MO BIO Laboratories, Inc. Carlsbad, California, United States). DNA concentration was determined by the Qubit^®^ 2.0 flowmeter (Life Technologies, Grand Island. New York, United States). Progressive molecular indicators (primer-sense/forward—515F; 5’-GTGCCAGCMGCCGCGGTAA-3′) and reverse (primer anti-sense/reverse—806R; 5’-GGACTACHVGGGTWTCTA AT-3′) that aims a 291 pb fraction from V4 region were utilized, for amplification (IDT Corp., Coralville, Iowa, United States) (29) of the 16S rRNA gene, using the Fluidigm Access Array technique (Fluidigm Corporation, South San Francisco, California, United States) in combination with the Roche High Fidelity Fast Start Kit (Roche, Indianapolis, Indiana, United States). The amplicons quality was evaluated using a fragment analyzer (Advanced Analytics, Ames, Iowa, United States) to confirm the regions and sizes of amplicons. Equimolar quantities of amplicons of each sample were mixed and the amplicon sizes were selected in agarose E-gel at 2% (Life Technologies, Grand Island, New York, United States) and extracted with a Qiagen gel purification kit (Qiagen, Valencia, California, United States). The sequencing, utilizing Illumina platform, was made in a MiSeq sequencer using v3 reagents (Illumina Inc., San Diego, California, United States) at the Illinois University Biotechnological Center. Fluidigm tags were removed using a FASTX-Toolkit (version 0.0.14) and the QIIME 1.9.1 software was used to process the resulting sequence data (30). High-quality sequences (quality value ≥ 20) were demultiplexed. The sequences were then grouped in operational taxonomic units (OTU) using an open reference OTU selection against the Greengenes database with a similarity level of 97%. Singletons (OTUs observed less than twice) and OTUs which represented less than 0.01% were excluded. A total of 1.353.878 sequences were obtained with a mean of 32,235 and interval = 19,737–57.888 sequences per sample. Rarefaction was performed for alpha and beta diversity analysis, maintaining 19,730 sequences per sample. Principal Component Analysis (PCA) was made using weighted and non-weighted unique metric distances of the fraction (UniFrac) (31).

### 2.6 Statistical analyses

The data were expressed as mean ± standard error of mean (SEM). Normality and homogeneity of variances were subjected to Shapiro–Wilk and Levene tests. The statistical analysis was performed using the MIXED procedure from SAS Studio statistical software (SAS Institute, Cary, NC, United States) with insect meal and inclusion levels as fixed effects and cats and period as random effects. A two-way ANOVA was made with insect meals and inclusion levels as factors. The Tukey test was carried out to compare the treatments at 5% probability. In case of differences detected only for insect meal, GLM procedure of SAS was performed followed by Tukey’s test at 5% probability. Pearson’s correlation was selected to perform correlation analysis of microbiota.

## 3 Results

The average daily intake (g) of insect meals for treatments with 7.5 and 15% of inclusion levels were, respectively, 4.44 and 8.14 for the CC meal, 4.32 and 8.26 for the MC meal, and 4.13 and 8.09 for the SW meal. Furthermore, the average dietary feed intake (g/d) reported were 65.85 and 60.43 for CC, 64.40 and 68.26 for MC, and 62.02 and 58.70 for SW, at 7.5 and 15% of inclusion, respectively.

There were no significant differences among insect meal diets on DM, OM and CP (*p* > 0.05), except EE that was higher in all diets with 15% of insect meal (*p* < 0.05) compared to the 7.5% diets. Also, the diet containing MC meal, regardless of inclusion level, presented the highest chitin content when compared to diets containing CC or SW meals (*p* < 0.05) (Table 2).

**Table 2:** Apparent digestible coefficients and apparent metabolizable energy of dry matter (AME DM) of the diets with three insect meals at two different inclusion levels for adult cats.

	Treatments^a^							*p* value		
Item	7.5% CC	15% CC	7.5% MC	15% MC	7.5% SW	15% SW	SEM	IM	IL	IM × IL
DM (%)	84.10	83.86	83.72	82.41	84.77	85.01	5.82	0.402	0.691	0.836
OM (%)	41.22	34.69	36.88	36.46	36.91	35.37	47.21	0.938	0.513	0.825
CP (%)	89.12	87.30	87.41	87.53	87.22	88.85	4.70	0.774	0.978	0.299
EE (%)	90.21b	92.96a	88.16b	90.20a	90.67b	92.61a	4.40	0.106	0.041	0.940
C (%)	30.89b	25.92c	32.06a	32.03a	31.54b	26.71c	11.33	0.002	0.002	0.021
AME DM (kcal/kg)	3,789	3,872	3,768	3,620	3,912	3,898	5.76	0.007	0.593	0.176

a Formulated diets containing different sources of insect meal at different ratios.

CC = Cinerea Cockroach; MC = Madagascar Cockroach; SW = Superworm, Dry Matter (DM), Organic Matter (OM), Crude Protein (CP), Ethereal Extract in Acid Hydrolysis (EE), Chitin (C) of meals and Apparent metabolizable energy of dry matter (AMEDM). SEM = standard error of the mean, IM = Insect meal, IL = Inclusion level, IM × IL = interaction between insect meal and inclusion level. Means followed by the same letter do not differ from each other by the Tukey test at 5% significance, as uppercase letters are for interaction of meal x level and lowercase letters are only the type of insect meal tested.

There were no significant effects of insect meal for ADC_DM_, ADC_OM_, ADC_CP_ and ADC_EE_ (*p* > 0.05). However, a significant difference was registered on the ADC of chitin (ADC_C_) (*p* < 0.05) for MC diets regardless of inclusion level and despite the interaction of these two factors did not present statistical significance (*p* > 0.05) (Table 3). Diets containing MC meal presented the highest ADC_C_ when compared to diets containing CC or SW meals.

**Table 3:** Apparent digestibility coefficients (ADC %) of diets with three insect meals at two different inclusion levels for adult cats.

	Treatments^a^							*p* value		
Item	7.5%CC	15%CC	7.5%MC	15%MC	7.5%SW	15%SW	SEM	IM	IL	IM × IL
ADC_DM_ (%)	84.99	85.04	84.94	84.94	85.08	85.12	1.8845	0.9964	0.9834	0.9999
ADC_OM_ (%)	85.71	85.60	85.57	85.52	85.09	85.69	1.5884	0.9858	0.9117	0.9699
ADC_CP_ (%)	87.14	86.86	86.91	86.88	86.88	86.97	1.4786	0.9972	0.9527	0.9924
ADC_EE_ (%)	92.03	92.36	91.76	92.17	92.09	92.33	1.1800	0.9732	0.7378	0.9972
AD_CC_ (%)	25.06d	25.93c	32.06a	32.04a	31.20b	26.67c	1.2111	0.0020	0.0147	0.0538
AME_DM_ (kcal/kg)	3960.83	3912.67	4012.83	3957.17	3883.17	3908.50	85.11	0.5827	0.7092	0.8714

a Formulated diets containing different sources of insect meal at different ratios.

CC = Cinerea Cockroach; MC = Madagascar Cockroach; SW = Superworm, Apparent digestibility coefficients (ADC) of Dry Matter (DM), Organic Matter (OM), Crude Protein (CP), Ethereal Extract in Acid Hydrolysis (EE), Chitin (C) of meals and Apparent metabolizable energy of dry matter (AMEDM). SEM = standard error of the mean, IM = Insect meal, IL = Inclusion level, IM × IL = interaction between insect meal and inclusion level. Means followed by the same letter do not differ from each other by the Tukey test at 5% significance, as uppercase letters are for interaction of meal x level and lowercase letters are only the type of insect meal tested.

Cats fed 15% MC registered higher urinary pH (*p* < 0.05) (Table 4). For urinary density analysis, no differences occur (*p* > 0.05) among treatments. The same occurs for fecal score, fecal pH, urea, creatinine, and blood count (red blood cells, leukocytes, lymphocytes, platelets), whereas no significant differences were registered (*p* > 0.05) (Table 4).

**Table 4:** Urinary, fecal and blood parameters of cats fed with different insect meals with different inclusion levels (7.5 and 15% of inclusion).

	Treatments^a^							*p* value		
Item	7.5%CC	15%CC	7.5%MC	15%MC	7.5%SW	15%SW	SEM	IM	IL	IM × IL
Urinary Density	1,024	1,021	1,023	1,027	1,020	1,021	0.69	0.043	0.668	0.096
Urinary pH	6.75B	6.61B	6.61B	7.01A	6.71B	6.74B	4.38	0.294	0.156	0.008
Fecal pH	6.10	6.01	6.10	6.15	6.12	6.22	7.09	0.778	0.874	0.853
Fecal score	3.83	3.83	3.83	3.83	4.00	3.66	12.77	0.999	0.372	0.449
Water consumption	129.66	134.17	129.66	107.17	139.50	124.17	41.99	0.370	0.220	0.443
Blood parameters										
Urea (mg/dL)	56.50	59.33	54.16	49.83	58.83	54.33	43.08	0.412	0.597	0.662
Creatine (mg/dL)	1.10	1.08	1.16	1.01	1.16	1.01	31.18	0.999	0.205	0.741
Red blood cells (mm^6^)	8.64	8.69	8.83	9.63	8.78	9.21	17.75	0.166	0.085	0.434
Leukocytes (mm^3^)	13.24	13.52	14.50	15.40	13.50	14.78	28.48	0.549	0.500	0.941
Lymphocytes (mm^3^)	24.33	28.33	24.33	22.16	25.17	25.50	43.35	0.578	0.768	0.587
Platelets (mil/mm^3^)	471.00	549.83	544.50	511.83	501.33	465.83	31.22	0.646	0.928	0.406

a Formulated diets containing different sources of insect meal at different ratios.

CC = Cinerea Cockroach; MC = Madagascar Cockroach; SW = Superworm. SEM = standard error of the mean, IM = Insect meal, IL = Inclusion level, IM × IL = interaction between insect meal and inclusion level. Means followed by the same letter do not differ from each other by the Tukey test at 5% significance, as uppercase letters are for interaction of meal x level and lowercase letters are only the type of insect meal tested.

No differences were registered (*p* > 0.05) for total fecal BSCFAs, total BCFAs, phenol and indole in faeces of cats fed different insect meals (Table 5). Cats fed MC meal presented low isovalerate and high 4-Methylphenol (*p* < 0.05).

**Table 5:** Branched short-chain fatty acid (BSCFAs), branched-chain fatty acids (BCFAs), phenol and indole in faeces of cats fed different insect meals.

	Treatments^a^						*p* value		
Item	7.5%CC	15%CC	7.5%MC	15%MC	7.5%SW	15%SW	IM	IL	IM × IL
Acetate (mg/g)	137.08	150.62	114.49	147.16	140.89	119.70	0.429	0.387	0.085
Propionate (mg/g)	45.91	48.86	40.83	49.82	47.14	40.02	0.690	0.657	0.204
Butyrate (mg/g)	26.79	26.31	22.13	28.97	24.58	21.3	0.628	0.746	0.407
Total BSCFA	209.78	225.79	177.45	225.95	212.61	181.02	0.082	0.064	0.074
Isobutyrate (mg/g)	3.35	3.44	2.57	2.90	3.30	2.38	0.205	0.601	0.245
Isovalerate (mg/g)	5.91b	6.34a	4.79c	4.58c	6.18a	4.52c	0.048	0.291	0.162
Valerate (mg/g)	12.08	11.18	9.18	9.82	12.53	9.22	0.354	0.331	0.410
Total BCFA	22.06	20.96	16.54	17.3	22.01	16.12	0.078	0.225	0.092
4-Methylphenol (mg/g)	266.76b	191.40c	325.80a	355.58a	279.97b	245.72b	0.016	0.375	0.356
Indole (mg/g)	22.33	80.66	51.40	47.76	51.12	57.81	0.979	0.304	0.394

a Formulated diets containing different sources of insect meal at different ratios.

CC = Cinerea Cockroach; MC = Madagascar Cockroach; SW = Superworm. SEM = standard error of the mean, IM = Insect meal, IL = Inclusion level, IM × IL = interaction between insect meal and inclusion level. Means followed by the same letter do not differ from each other by the Tukey test at 5% significance, as uppercase letters are for interaction of meal x level and lowercase letters are only the type of insect meal tested.

**Figure 1:**
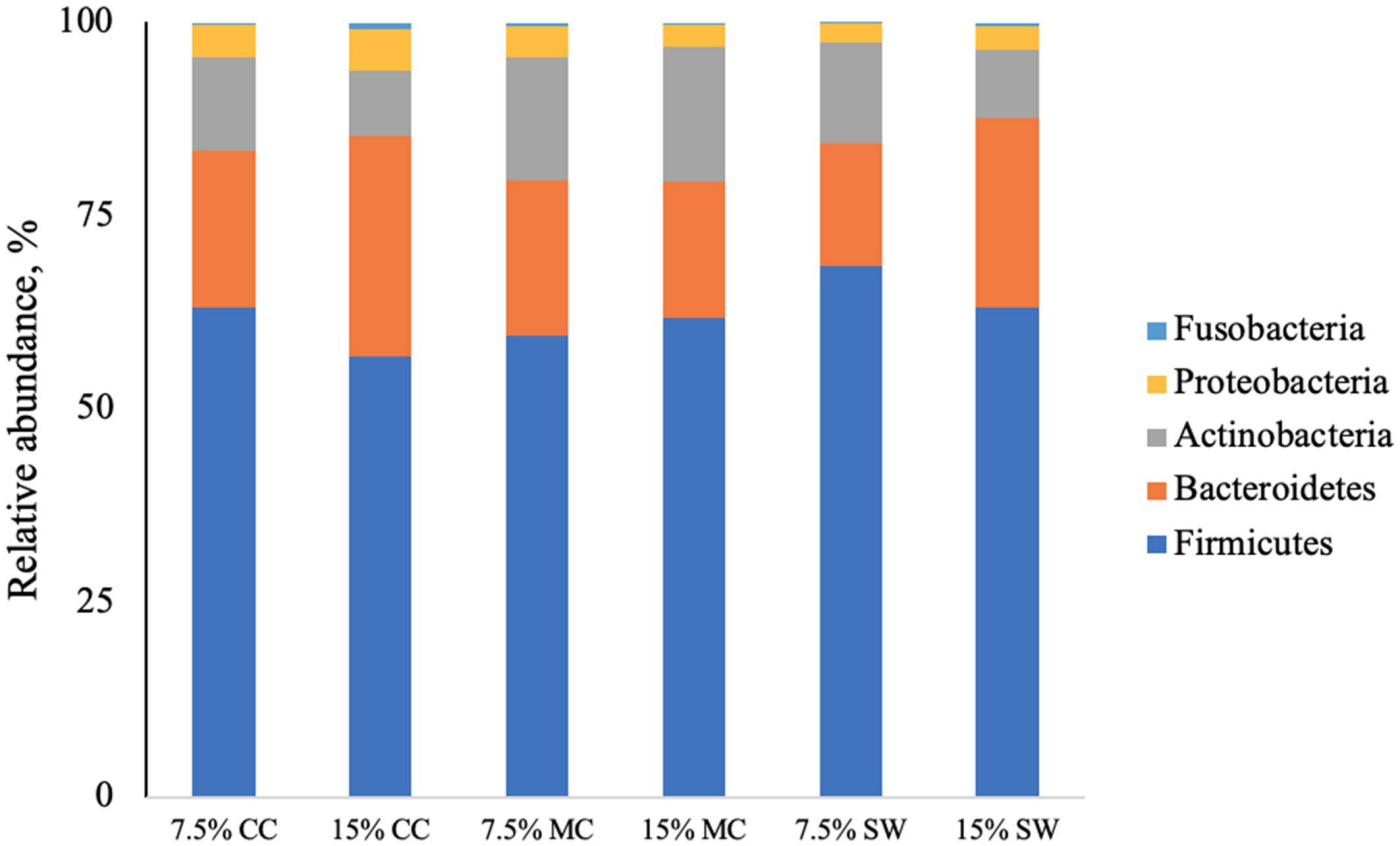
Relative abundance of bacterial phyla found in the feces of cats fed different levels of insect meal inclusions (Cinereal cockroach meal, Madagascar cockroach meal and Tenebrio meal). RD = reference diet, CC = Cinerea cockroach, MC = Madagascar cockroach, SW = Superworm.

**Figure 2:**
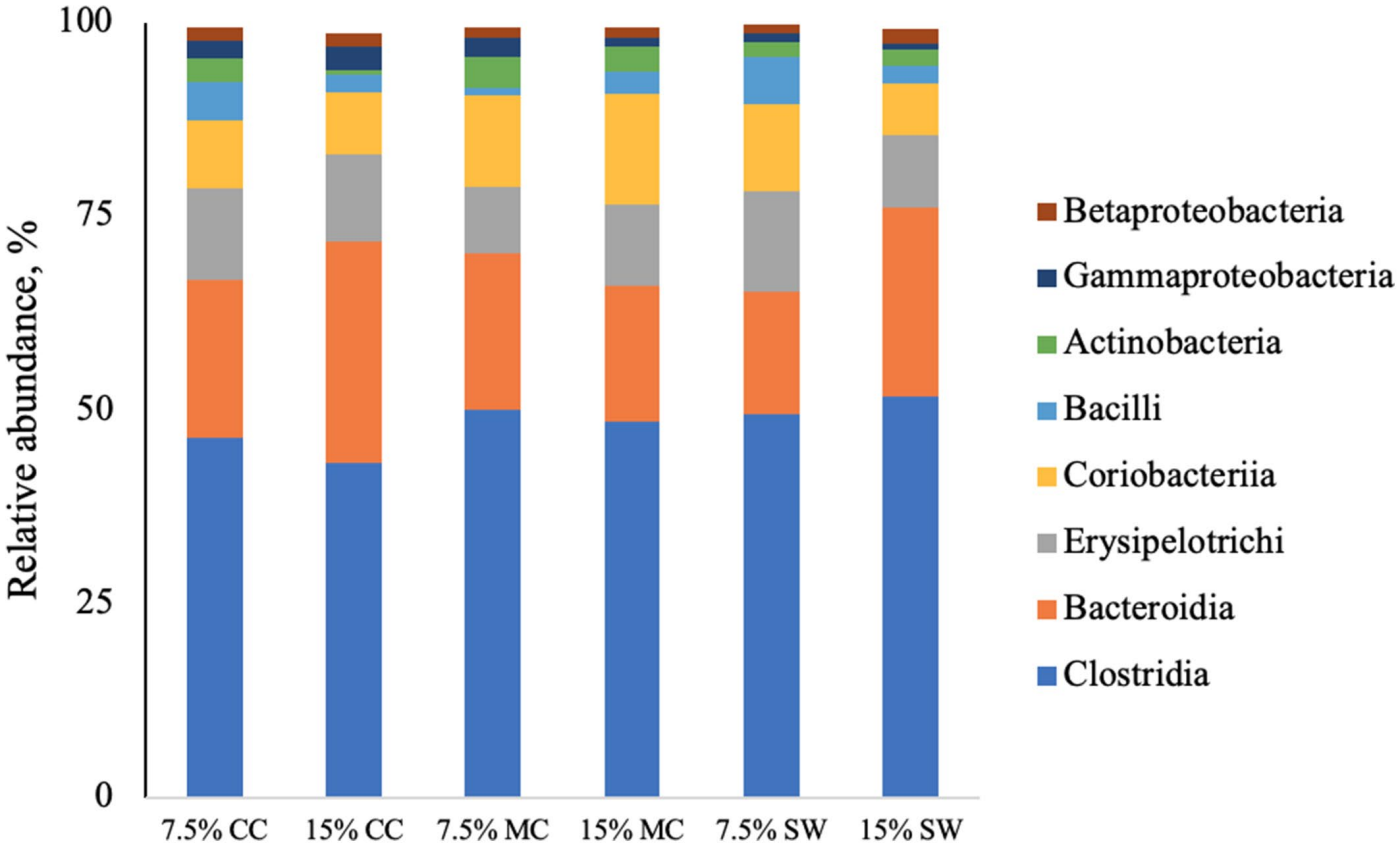
Relative abundance of bacterial classes found in the feces of cats fed different levels of insect meal inclusions (Cinereal cockroach meal, Madagascar cockroach meal and Tenebrio meal). RD = reference diet, CC = Cinerea cockroach, MC = Madagascar cockroach, SW = Superworm.

**Figure 3:**
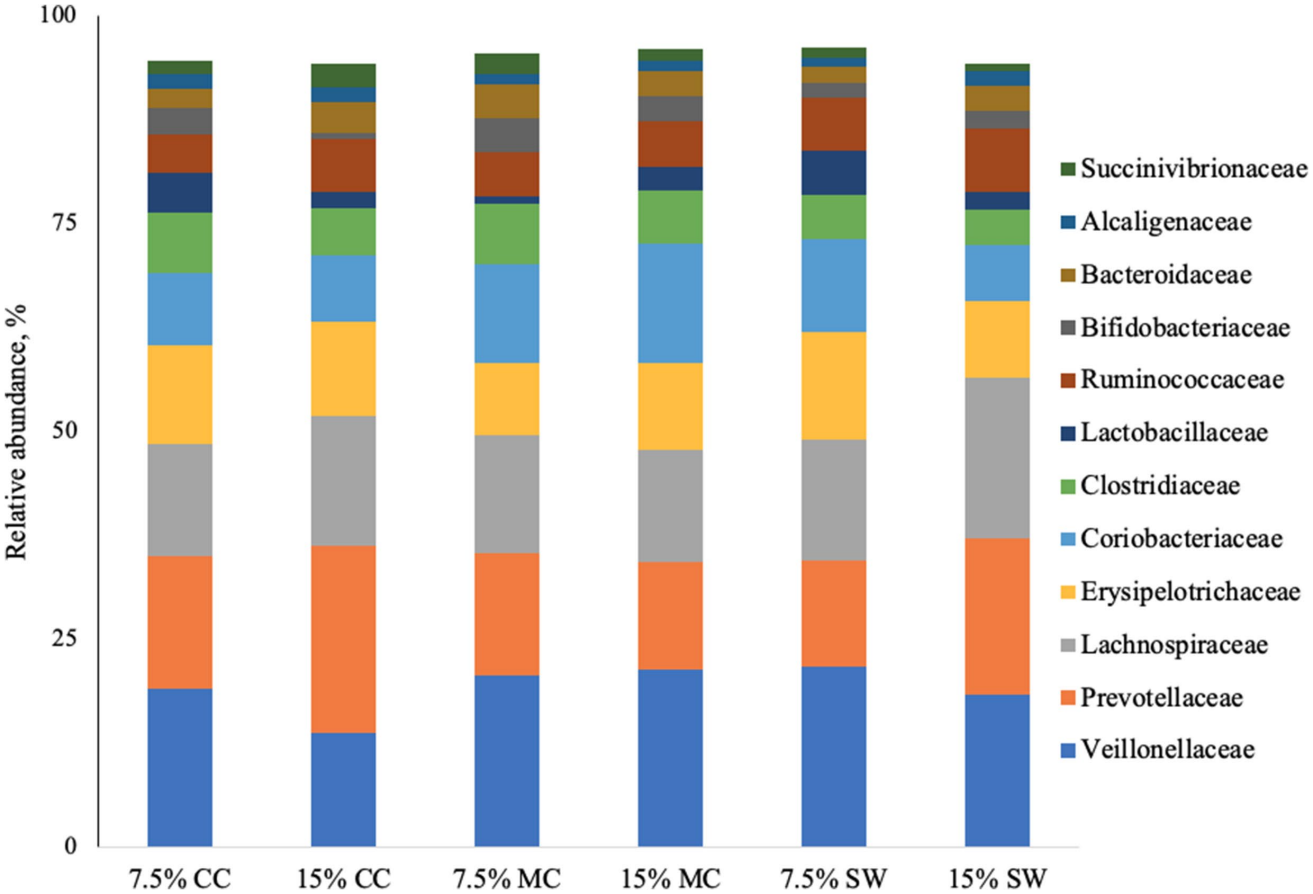
Relative abundance of bacterial families found in the feces of cats fed different levels of insect meal inclusions (Cinereal cockroach meal, Madagascar cockroach meal and Tenebrio meal). RD = reference diet, CC = Cinerea cockroach, MC = Madagascar cockroach, SW = Superworm.

**Figure 4:**
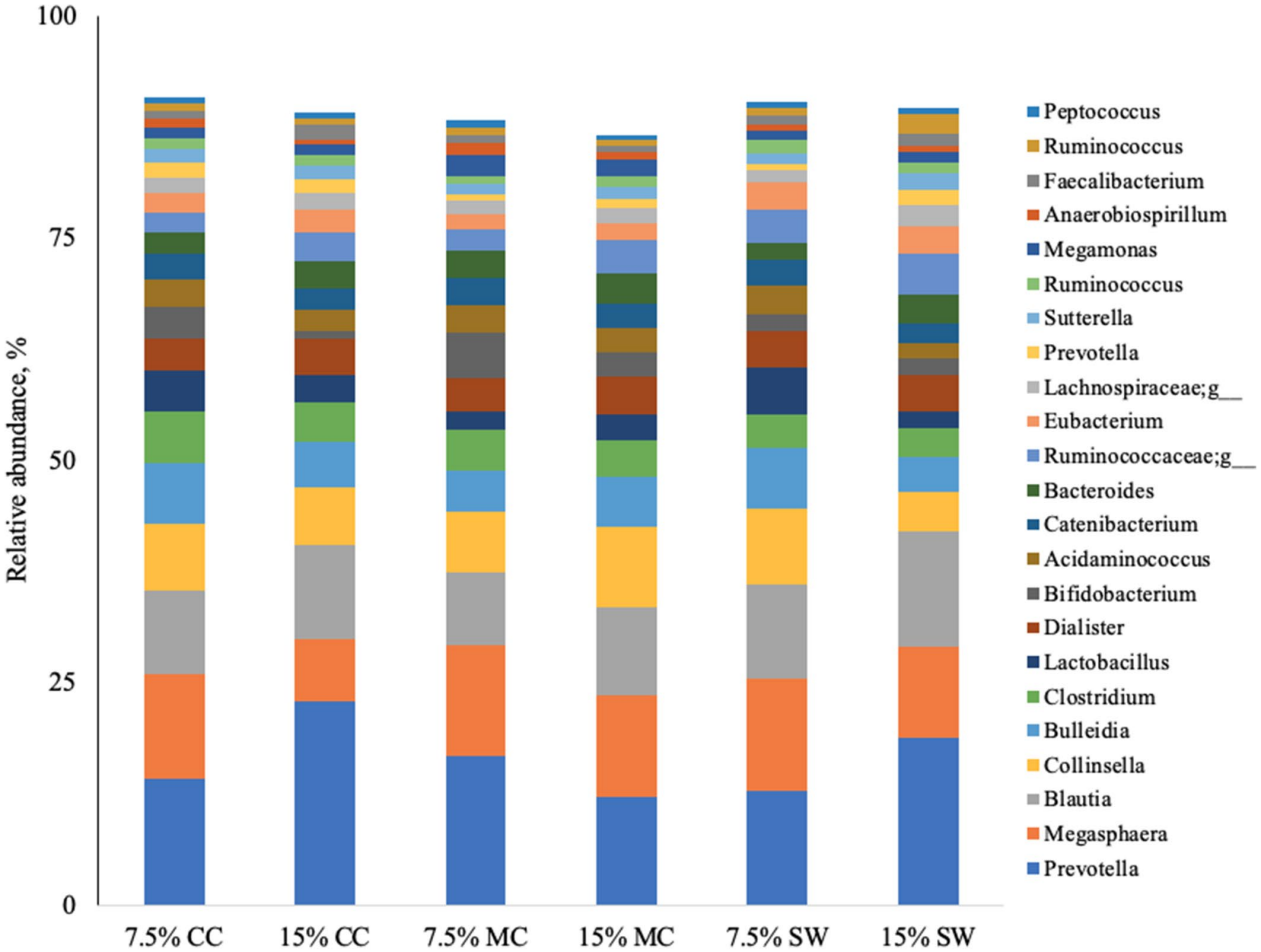
Relative abundance of bacterial genera found in the feces of cats fed different levels of insect meal inclusions (Cinereal cockroach meal, Madagascar cockroach meal and Tenebrio meal). RD = reference diet, CC = Cinerea cockroach, MC = Madagascar cockroach, SW = Superworm.

The main phyla found in all cat samples were *Firmicutes* (57–68%) followed by *Bacteriodetes* (11–29%), *Actinobacteria* (9–22%), P*roteobacteria* (1–5%) and F*usobacteria* (0–1%) (Figure 1). A*ctinobacteria* phylum was a prominent taxonomic group in cats when fed the reference diet while *Bacteroidetes* and *Proteobacteria* phyla were present in higher abundance in cats fed insect meal. *Clostridia* was the most relatively abundant taxa in all groups. *Bacteroida* was present in higher percentage on cats fed insect meal while cats fed control diet showed higher percentages of *Coriobacteria* and *Actinobacteria* classes (Figure 2). The higher percentage of *Firmicutes* phylum in faeces of cats fed the reference diet were from the families *Veillonellaceae*, *Coriobacteriaceae* and *Lactobacillaceae* (Figure 3). Cats fed insect meal presented higher percentages of sequences of *Prevotellaceae* followed by *Veillonellaceae* and *Lachnospiraceae* (Figure 4).

Of the 27 most abundant taxa at the genus level or higher which comprised 90% of sequences reads, 15 were significantly affected by insect meal. *Prevotella* (*Bacteroidetes*) was a prominent taxonomic group across cats fed insect meal while the genus *Megasphaera* (*Firmicutes*) was more prevalent in cats when fed the reference diet (Figure 4). Cats fed 15% CC and 15% MC presented the highest percentage of *Prevotella* and *Blautia* (*Firmicutes*) when compared to cats fed other insect meals or the reference diet.

The analysis of alpha and beta-diversity indices revealed that the fecal microbial communities of cats fed diets containing different insect meal and inclusion levels exhibited a remarkable similarity. These findings indicated that there were no significant differences observed in the microbial composition among the treatments or even when compared to the reference diet.

## 4 Discussion

The present study demonstrates that larvae meal from the Madagascar cockroach (*Gromphadorhina portentosa*), the Cinerea cockroach (*Nauphoeta cinerea*), and the Superworm (*Zophobas morio*) can be considered an alternative protein source in cat nutrition. Although the insects used in this trial were from different orders, the data indicated no differences in the apparent digestibility of DM, OM, and CP. Unaffected or even improved nutrient digestibility may vary according to the animal species, potentially due to specific abilities in chitin digestion and the processing of other fibers present in the diet.

Chitin, a natural polymer found in the exoskeletons of insects, may inhibit the absorption of lipids and proteins, thereby affecting the digestibility coefficient of insect meals (32, 33). In this study, the chitin content ranged from 1.68 to 5.05%. However, chitin did not influence lipid digestibility, likely due to its high digestibility, as reported by Fontes et al. (12) in studies on insect digestibility in fish feeds. Chitin content may interfere with the dietary utilization of protein, with a reduction in protein digestibility expected as chitin content increases (33).

A study carried out with ten domestic cats using black soldier fly larvae (*Hermetia illucens*) meal at 22 and 35% inclusion showed a high DM content (95.6%) with moderate protein digestibility ranging from 69 to 80% and crude fat apparent digestibility between 91 and 97% (34). The authors attributed the moderate crude protein digestibility to the extrusion process and the interaction of intestinal fibers already present in the meal. Differences in the ADC of insect meals may be attributed to various factors, including insect rearing conditions, processing methods, and nutritional composition between insect species (35). Biasato et al. (36) showed that piglets fed 5% or 10% black soldier fly larvae meal had ADC values of 95.5 and 95.9% for DM content, 80.8 and 82.8% for CP, and 85.7 and 85.6% for EE content, respectively.

Urinary pH in felines can be directly affected by feed composition, including nutrient profile and feed volume, among other factors (37). This may explain the significant increase in urinary pH observed in cats fed the diet containing 15% Madagascar cockroach meal. Notably, this effect was not observed in the other insect meal treatments when compared to the control group, suggesting that the urinary pH alteration may be specifically related to the level of inclusion or to particular components of the Madagascar cockroach meal. It is possible that some nutritional or chemical characteristics of this ingredient, such as a higher mineral content or buffering capacity, may have reduced urinary acidification. Further investigation is warranted to better understand this response. Funaba et al. (37) reported no significant differences in the urinary pH of cats fed fish meal and corn gluten (pH values of 6.11 and 6.14, respectively), similar to the results found in this study for cats fed CC and SW meals. The authors highlighted that protein sources did not influence urine acidification, corroborating the findings of Skoch et al. (38), who reported similar urinary pH values (between 6.3 and 6.4) for meat and bone or corn gluten meals. Paßlack et al. (34) showed stable pH values (8.3 and 8.49) in cats fed different qualities and percentages of protein (36.2–56.1%). The fecal score was within established standards for cats fed dry diets, with highly digestible feed resulting in well-formed solid faeces (39). Although the inclusion of insect meals influenced the ADC of chitin, no changes in fecal score or quality were observed, maintaining values between 3 and 4.

The hematological parameters such as proteins, erythrogram, and glucose can be influenced by dietary protein (40, 41). In the current study, all cats exhibited circulating red blood cells, leukocytes, lymphocytes, and platelet counts within the reference values for healthy cats. Similar results were reported in a previous study with dogs using the same insect meal and inclusion levels (18). For instance, in Nile tilapia (*Oreochromis niloticus*), the inclusion of 15 and 30% superworm meal in diets did not affect the total leukocyte count compared to fish on a basal diet, as shown by Alves et al. (11). Similarly, in pigs fed black soldier fly larvae as a replacement for fish meal at different levels (0, 25, 50, 75, and 100%), no significant differences were observed in red or white blood cell parameters, except for neutrophil counts, which were higher at 75 and 100% replacement, as reported by Chia et al. (42). It is worth noting that results concerning blood parameters may vary depending on the insect meal used and the animal species involved in the studies.

The compounds responsible for fecal odor can be categorized into five groups: phenol, indole, branched-chain fatty acids, amines, and sulfur compounds. The concentration of these compounds can be influenced by dietary protein content and the production of amino acids during metabolism. In this study, no significant differences were observed in the production of phenol and indole. This finding is desirable, as phenol and indole have the potential to interact with other putrefaction compounds in the intestine, potentially enhancing their carcinogenic effects by acting as cocarcinogens (43, 44).

The dietary inclusion of insect meals did not affect the total production of BSCFAs. However, the greater ratio of propionate in the faeces of all cats fed insect meals may indicate that carbohydrate fermentation in the hindgut was modified by the treatments, as reported in laying hens fed an insect-based diet (45). The fermentative rate and BSCFA production depend on the quality of substrates provided by the diet (46). According to Louis et al. (47), many bacterial species in the intestine can produce acetate, butyrate, and propionate from lactate.

The intestinal microbiota is associated with many metabolic functions and plays a role in maintaining a healthy gastrointestinal tract. Fecal microbiota analysis suggests that the inclusion of insect meals up to 15% does not affect the microbial community in terms of the abundance and presence of different taxa. The microbial community can change in different parts of the body and along the gastrointestinal tract (48, 49). Basic functions are conserved independently of the microbial niche, suggesting that a specific group of microorganisms is responsible for maintaining a favorable symbiotic system. Disruption of this microbial group can lead to dysbiosis and potentially cause physiological imbalances and pathogenic processes in the host (50).

Previous studies have identified Firmicutes, Bacteroidetes, Proteobacteria, Fusobacteria, and Actinobacteria as the predominant phyla in the gastrointestinal tracts of healthy cats (51, 52). The fecal microbiota results of this study are consistent with these findings. While there is a limited amount of research on the microbiomes of pet animals, it is known that dietary interventions can modulate intestinal microbiota in cats and dogs (53, 54). Traditionally, studies investigating microbiota modulation have focused on dietary fibers and prebiotics, which promote beneficial microbial populations such as Actinobacteria and Bifidobacterium (55–57). For instance, Butowski et al. (51) demonstrated that diets rich in raw meat increased the abundance of Firmicutes and Fusobacterium, while combining raw meat with fibers promoted higher levels of beneficial bacteria like Bifidobacterium and Actinobacteria. Other studies have examined the effects of moderate and high protein concentrations in diets for young cats (58, 59). According to Deusch et al. (58), a higher protein diet (> 50% DM) results in greater diversity and specialization of fecal microbiota.

Additionally, a lower proportion of sequences corresponding to the *Lactobacillaceae* family and an increase in P*revotellaceae* were observed. Bermingham et al. (60) reported that *Lactobacillus* and *Bifidobacterium*, which are generally considered beneficial to the host, dominate in younger kittens (18 weeks), while *Bacteroidetes* and *Prevotella* (*Prevotellaceae*) are the dominant genera in adult cats (42 weeks), which aligns with our findings. However, given the lack of studies evaluating the effects of insect larvae meal supplementation in cat food, further research is needed to isolate and characterize the compounds in insect meals that modulate feline microbiota and to explore the interactions between age, diet, and changes in gastrointestinal microbiota.

This study found no significant impact of the diets on the intestinal microbiota of cats. This conclusion is supported by the lack of distinct clustering patterns in the fecal microbiota, as observed in the PCA graphics and rarefaction curves. These results indicate that there were no significant changes in species richness or alterations in the composition and relative abundance of microbial communities within the intestines of cats fed different insect meals. Similar findings were reported in a previous study with dogs using the same insect meal and inclusion levels (18), as well as in cats (61). Notably, the use of insect meals did not induce dysbiosis in the intestinal microbiota of cats or adversely affect their health, as reflected in the microbiota profile, abundance, and blood parameters evaluated. Furthermore, previous research has suggested the potential prebiotic properties of chitin in the intestinal environment (18, 45). The observed alterations in the microbiota over a relatively short period in our study highlight the potential prebiotic effects of insect meals on feline intestinal health.

## 5 Conclusion

This study highlights the potential of insect larvae meals, such as Madagascar cockroach, Cinerea cockroach, and superworm, as viable alternative protein sources in adult cat diets. The findings indicate that these diets do not significantly alter intestinal microbiota, suggesting no adverse effects on species richness or microbial composition. Additionally, the presence of chitin in insect meals may offer prebiotic benefits, further supporting their positive influence on gastrointestinal health. Diets containing up to 15% insect larvae meal are a suitable option for cat nutrition, providing a promising alternative to traditional protein sources. However, more research is needed to confirm these benefits and to assess how various insect proteins may affect the digestive systems of cats at different life stages. Additionally, further studies are required to evaluate key aspects such as palatability, digestibility, allergenicity, and long-term safety of incorporating insects into feline diets.

## Data Availability

The original contributions presented in the study are included in the article/supplementary material, further inquiries can be directed to the corresponding author.
